# *Cryptosporidium parvum* infection attenuates the ex vivo propagation of murine intestinal enteroids

**DOI:** 10.14814/phy2.13060

**Published:** 2016-12-30

**Authors:** Xin‐Tian Zhang, Ai‐Yu Gong, Yang Wang, Xiqiang Chen, Sheng‐Yau S. Lim, Courtney E. Dolata, Xian‐Ming Chen

**Affiliations:** ^1^ Department of Medical Microbiology and ImmunologyCreighton University School of MedicineOmahaNebraska

**Keywords:** *C. parvum*, cryptosporidiosis, cytokines, enteroids, intestine, Lgr5, mice, neonatal, stem cells

## Abstract

*Cryptosporidium,* a ubiquitous coccidian protozoan parasite that infects the gastrointestinal epithelium and other mucosal surfaces, is an important opportunistic pathogen for immunocompromised individuals and a common cause of diarrhea in young children in the developing countries. One of the pathological hallmarks of intestinal cryptosporidiosis is villous atrophy, which results in a shorter height of intestinal villi. Here, we investigated the effects of *Cryptosporidium* infection on intestinal epithelial growth, using an ex vivo model of intestinal cryptosporidiosis employing enteroids from mice. We detected infection of enteroids isolated from immunocompetent adult and neonatal mice after ex vivo exposure to *Cryptosporidium* sporozoites. We observed a significant inhibition of enteroid propagation following infection. Intriguingly, we identified a decreased expression level of intestinal stem cell markers in enteroids following *C. parvum* infection. We further measured the expression levels of several Wnt antagonists or agonists in infected enteroids, as induction of the Wnt/*β*‐catenin activation is a key factor for intestinal stem cell function. We detected a markedly increased level of the Dickkopf‐related protein 1 and decreased level of the Wnt family member 5a in enteroids after infection. The low density lipoprotein receptor‐related protein 5, one of the Wnt co‐receptors, is downregulated in the infected enteroids. In addition, increased apoptotic cell death and cell senescence were observed in the infected enteroids. Our results demonstrate a significant inhibitory effect of *Cryptosporidium* infection on the ex vivo propagation of enteroids from mice, providing additional insights into the impact of *Cryptosporidium* infection on intestinal epithelial growth.

## Introduction

*Cryptosporidium*, a ubiquitous coccidian protozoan parasite, infects the gastrointestinal epithelium and other mucosal surfaces, causing an asymptomatic or self‐limited disease in immunocompetent individuals but a life‐threatening diarrheal disease in AIDS patients (Checkley et al. 2015). *Cryptosporidium* is also a cause of diarrhea in children worldwide and is one of the most common pathogens responsible for moderate‐to‐severe diarrhea in children in the developing regions, particularly in infants (Kotloff et al. 2013). Severe cryptosporidiosis is closely associated with mortality and children who survive infections can suffer from lasting growth and developmental defects (Pierce and Kirkpatrick 2009; Putignani and Menichella 2010; Striepen 2013). Despite its significant morbidity, mortality, and cost to society, there is currently no fully effective therapy available (Chen et al. 2002; Striepen 2013).

The majority of human cryptosporidial infections are caused by two species: *C. parvum* and *C. hominis* (Chen et al. 2002). *C. parvum* sporozoites attach to the apical membrane of intestinal epithelial cells (mainly villus enterocytes) and form a parasitophorous vacuole in which the organism remains intracellular but extracytoplasmic, limiting a direct infection often only to enterocytes and preventing a direct infection of immune cell types (Chen et al. 2002). Due to this “minimally invasive” nature of infection, intestinal epithelium provides the first line of defense and plays a critical role in activating and orchestrating host responses to *C. parvum* infection (Chen et al. 2002). Indeed, the invasion of enterocytes by *C. parvum* activates the nuclear factor‐kappa B (NF‐ĸB) signaling, resulting in the production and secretion of various cytokines and chemokines, antimicrobial peptides (*β*‐defensins and cathelicidins), and nitric oxide, which may kill *C. parvum* or inhibit parasite growth (Laurent et al. 1998; O'Hara and Chen 2011; Zhou et al. 2012). In addition, infection increases release of epithelial cell‐derived exosomes to the lumen and to the basolateral region (Hu et al. 2013). Subsequently, these chemokines/cytokines of enterocyte origin, as well as epithelial cell‐derived exosomes, mobilize and activate immune effector cells (e.g., NK cells, macrophages, dendritic cells, CD4,^+^ and CD8^+^ lymphocytes) at the site of infection (Chen et al. 2002).

One of the pathological hallmarks of intestinal cryptosporidiosis is villous atrophy (villi became stunted and shortened), with a diffuse shortening or loss of brush border microvilli (Farthing 2000; Huang and White 2006). The imbalance of absorption and secretion is likely a major contributor to disease manifestation, such as diarrhea. Infection may have negative effects on intestinal epithelial growth, contributing to the pathogenesis of intestinal cryptosporidiosis. The intestinal epithelium exhibits a remarkable capacity of self‐renewal, renewed rapidly every 3–5 days in human, to maintain intestinal homeostasis (Barker et al. 2010); this property reflects the activity of multipotent intestinal stem cells (ISCs) which divide and later differentiate into all intestinal subtypes (enterocytes, goblet cells, Paneth cells, and neuroendocrine cells) in the intestinal epithelium (Barker 2014). Current understanding of the impact of *Cryptosporidium* infection on intestinal epithelial growth remains very limited. Its potential influence on ISCs located in the crypts has yet to be explored. Nevertheless, infection models for such pathophysiological study are limited because immunocompetent adult mice are naturally resistant to infection (Kim 1994).

Enteroids are functional 3D cultured intestinal epithelial units that recapitulate integral aspects of the intestine (Zachos et al. 2016). These enteroids contain multiple intestinal epithelial cell types that comprise the intestinal epithelium (enterocytes, goblet, enteroendocrine, Paneth, and stem cells) and are physiologically active based on responses to agonists (Zachos et al. 2016). They have been successfully utilized as models for pathogenic infections, including infection by bacteria and viruses (Foulke‐Abel et al. 2014). Previous studies indicate that intact crypts isolated from human intestines growing in culture medium supplemented with growth factors and antiapoptotic molecules support *Cryptosporidium* better than existing cell lines (Castellanos‐Gonzalez et al. 2013). In the work described here, we tested the effects of *C. parvum* infection on the ex vivo development of enteroids from immunocompetent mice. Infection and associated gene expression were confirmed in enteroids isolated and propagated from adult and neonatal mice. We observed a significant inhibition of enteroid propagation following infection. Intriguingly, we identified decreased expression levels of intestinal stem cell markers, the leucine rich repeat containing G protein coupled receptor 5 (Lgr5) and the sex determining region Y‐box 9 (Sox9), in the enteroids during *C. parvum* infection. A significant increased level of Dickkopf‐related protein 1 (Dkk1, a Wnt antagonist) and decreased levels of Wnt family member 5a (Wnt5a, a Wnt agonist) and low density lipoprotein receptor‐related protein 5 (Lrp5, a Wnt co‐receptor) were detected in the enteroids after infection. Increased apoptotic cell death and cell senescence was observed in the infected enteroids. Moreover, similar results were also observed in enteroids from in vivo infected neonatal mice through oral administration. Together, our findings indicate that *C. parvum* infection causes a significant inhibition on the growth of intestinal enteroids, presumably through multiple mechanisms involving inhibition of Wnt‐mediated ISC function, apoptotic cell death and cell senescence.

## Materials and Methods

### Ethics statement

This study was carried out in strict accordance with the recommendations in the Guide for the Care and Use of Laboratory Animals of the National Institutes of Health under the Assurance of Compliance Number A3348‐01. All animal experiments were done in accordance with procedures (protocol number #0960 and #0992) approved by the Institutional Animal Care and Use Committee of Creighton University.

### Intestinal villus/crypt isolation and enteroid propagation

Intestinal villus/crypt components from adults or neonatal mice were isolated with minor modification of the procedure outlined by previous studies (Liu et al. 2012) and all steps were carried out in buffers kept on ice. Briefly, 2.0 cm small intestine tissues above the cecum were collected from adult mice (1.5 cm for neonatal mice) and were opened longitudinally. Collected tissues were then put in 15 mL tube with 10 mL cold phosphate‐buffered saline (PBS) and inverted gently several times to wash the intestinal contents. Obtained tissues were further cut into 1~2 mm pieces and transferred into 15 mL tube containing 10 ml cold PBS with 2 mmol/L ethylene‐diamine‐tetraacetic acid, rotated for 30 min at 4°C, shaken vigorously until it became mostly opaque with dislodged crypt and villus particles. The villus/crypt mix was transferred to 50 mL tubes containing 10 mL cold PBS, filtered through a 70‐*μ*m cell strainer (MIDSCI, Cat. 258368‐70). The villus/crypt pellets were collected after centrifugation at 200g, 4°C, for 5 min, washed with 20 mL cold PBS and centrifuged one more time under the same condition. The pellets were then resuspended with cold PBS/Matrigel media (1:1 ratio in volume) and plated in 35 mm dishes (30 *μ*L mix per spot, 12 spots) or 60 mm dishes (30 spots). Enteroid culture medium (2 mL for 35 mm dishes and 4 mL for 60 mm dishes) were added and culture medium was changed every other day. The content of enteroid 3D culture medium was: epidermal growth factor (EGF, 500 *μ*g/mL) 50 *μ*L, Noggin (25 *μ*g/mL) 415 *μ*L, R‐Spondin 1 (250 *μ*g/mL) 200 *μ*L, B 27 Supplement 2 mL, N2 Supplement 1 mL, gentamicin (10 mg/mL) 500 *μ*L, 4‐(2‐hydroxyethyl)‐1‐piperazineethanesulfonic acid buffer (1 mol/L) 1 mL, Glutamax Supplement (100X) 1 mL, with the Dulbecco's Modified Eagle Medium:Nutrient Mixture F‐12 (DMEM/F‐12) to a final volume of 100 mL. For enteroid propagation, EGF, Noggin, R‐Spondin 1, and N2 supplement were added immediately before usage of the culture medium.

### *C. parvum* and infection in vivo and ex vivo

*Cryptosporidium parvum* oocysts harvested from calves inoculated with a strain originally obtained from Dr. Harley Moon at the National Animal Disease Center (Ames, IA) were purchased from a commercial source (Bunch Grass Farms, ID). Oocysts were purified using a modified ether extraction technique and then suspended in PBS and stored at 4°C. For oral gavage, oocysts were treated with 1% sodium hypochlorite on ice for 20 min and washed 3× with DMEM culture media. C57BL/6J mice at the age of 5–7 days after birth were used, as previously reported (Novak and Sterling 1991; Lacroix et al. 2001). Briefly, mice received *C. parvum* oocysts by oral gavage (10^5^ oocysts per mice). The mice receiving vehicle (PBS) by oral gavage was used as control. At 24 and 48 h after *cryptosporidium* or vehicle administration, animals were sacrificed and ileum intestine tissues were collected. At least five animals were sacrificed for each group. For ex vivo infection of cultured enteroids, oocysts were treated with 1% sodium hypochlorite on ice for 20 min and subjected to an excystation solution consisting of 0.75% taurodeoxycholate and 0.25% trypsin for 30 min at 37°C. The excystation rate was calculated as previously described by others (Chen et al. 2007) and was determined for each new batch of oocysts. These freshly excysted infective sporozoites were collected and added to the cultures for ex vivo infection. Infection was quantified by indirect immunofluorescence and real‐time PCR analysis of *C. parvum* 18s RNA, as previously reported (Zhou et al. 2012).

### Real‐time quantitative PCR

For quantitative analysis of mRNA expression, comparative real‐time PCR was performed using the SYBR Green PCR Master Mix (Applied Biosystems). RNA was extracted using TRI‐reagent, treated with the DNA‐free^™^ Kit (Ambion). RNA was reverse‐transcribed, using M‐MLV Reverse Transcriptase (Invitrogen). The following primers were used: *C. parvum* 18S ribosomal RNA (Cp18s) (forward, 5′‐TTGTTCCTTACTCCTTCAGCAC‐3′ and reverse, 5′‐TCCTTCCTATGTCTGGACCTG‐3′), mouse chemokine C‐X‐C motif ligand 2 (Mip‐2) (forward, 5′‐CACTCTCAAGGGCGGTCAAA‐3′ and reverse, 5′‐AGGCACATCAGGTACGATCCA‐3′), mouse nitric oxide synthase 2 (Nos2) (forward, 5′‐ATTTGGGAATGGAGACTGTC‐3′ and reverse, 5′‐CTGAAGGTGTGGTTGAGTTCT‐3′), mouse Dkk1 (forward, 5′‐TCCAACGCGATCAAGAACCT‐3′ and reverse, 5′‐CTCATCTTCAGCGCAAGGGTA‐3′), mouse intercellular adhesion molecule 1 (Icam1) (forward, 5′‐GCTGTTTGAGCTGAGCGAGAT‐3′ and reverse, 5′‐CGGAAACGAATACACGGTGAT‐3′), mouse interleukin 6 (IL‐6) (forward, 5′‐CCCAATTTCCAATGCTCTCCT‐3′ and reverse, 5′‐CATAACGCACTAGGTTTGCCG‐3′), mouse Lrp5 (forward, 5′‐ AACCGCGAGCCATTGTGTT‐3′ and reverse, 5′‐CCCATCTAGGTTGGCGCATT‐3′), mouse Wnt5a (forward, 5′‐AATCCACGCTAAGGGTTCC‐3′ and reverse, 5′‐TACAGGCTACATCTGCCAGG‐3′), mouse Wnt family member 3a (Wnt3a) (forward, 5′‐ATGGTCTCTCGGGAGTTTG‐3′ and reverse, 5′‐CCAGCAGGTCTTCACTTCA‐3′), mouse Lgr5 (forward, 5′‐CCTACTCGAAGACTTACCCAGT‐3′ and reverse, 5′‐GCATTGGGGTGAATGATAGCA‐3′), Sox9 (forward, 5′‐GCACTCTGGGCAATCTCA‐3′ and reverse, 5′‐GCTCAGTTCACCGATGTCC‐3′), mouse cyclin‐dependent kinase inhibitor 2A (p16) (forward, 5′‐TGAGAAGAGGGCCGCACCGGAATC‐3′ and reverse, 5′‐GCACCGGGCGGGAGAAGGTAGTG‐3′). Real‐time PCR was performed in triplicate. The Ct values were analyzed using the comparative Ct (ΔΔCt) method and the amount of target was obtained by normalizing to the endogenous reference (glyceraldehyde‐3‐phosphate dehydrogenase, GAPDH) and relative to the control (nontreated cells) (Chen et al. 2007; Zhou et al. 2012).

### Immunofluorescence

Cell cultures were fixed [0.1 mol/L, piperazine‐1,4‐bis(2‐ethanesulphonic acid) (Sigma‐Aldrich), pH 6.95, 1 mmol/L ethylene glycol‐bis (2‐oiminoether)‐ N,N,N′,N′‐tetraacetic acid] (Sigma‐Aldrich), 3 mmol/L magnesium sulphate (Sigma‐Aldrich) and 2% paraformaldehyde] at room temperature for 35 min and then permeabilized with 0.1% (v/v) Triton X‐ 100 in PBS. Fixed cultures were then incubated with primary antibodies and secondary antibodies, according to the standard immunochemical approach (Chen et al. 2007). Labelled cultures were then mounted using ProLong Gold Antifade Reagent with 4, 6‐diamidino‐2‐phenylindole (DAPI) stain (Invitrogen), followed by fluorescence microscopy. Anti‐Lgr5 (5 ng/mL, Upstate) and anti‐*C. parvum* (Zhou et al. 2012) were used. For localization of actin, fluorescein‐phalloidin (Sigma‐Aldrich) was incubated for 30 min at room temperature after incubation with the primary antibodies.

### Apoptosis and senescence assays

Apoptosis was quantitated by Annexin V binding. Cells were stained with the nuclear staining dye DAPI (2.5 *μ*mol/L, 5 min) or fluorescein‐labeled Annexin V (Pharmingen) and viewed with a fluorescence microscope. Senescent cells were detected using the Senescence‐associated *β*‐galactosidase (SA‐*β*‐gal) staining. Cultures were fixed and stained with the SA‐*β*‐gal cellular senescence assay kit (Cell Biolabs, San Diego, CA) following the manufacturer's protocol and as previously described (Kim et al. 2009). Senescence was further evaluated by detecting the induction of p16, a marker for senescence (Bernardes de Jesus and Blasco 2012). Expression levels of p16 were quantified by real‐time PCR as described above.

### Statistical analysis

All values are given as mean ± S.E. from at least three independent experiments and were compared with Student's *t* test (unpaired) or the Chi‐Square (2‐sided) test when appropriate. *P* < 0.05 were considered statistically significant.

## Results

### Infection of enteroids from adult mice by *C. parvum*

Since *C. parvum* infection is mainly localized in the ileum region in the intestine (Farthing 2000), we isolated the crypt/villus units from the small intestine within 2.0 cm above the cecum for adult mice (and 1.5 cm for neonatal mice) to develop enteroids for ex vivo infection. For enteroid propagation, as previously reported (Mahé et al. 2013), these isolated crypt/villus units were cultured in the defined medium in the 3D gel culture dishes, and developed into enteroids lined with a monolayer of cells showing sub‐apical membrane staining of actin typical to polarized intestinal epithelium, consistent with results from previous studies (Gao and Kaestner 2010; Mahé et al. 2013). For these isolated crypt/villus units or propagated enteroids, no obvious morphological difference was observed between these from adult mice and from neonatal mice (data not shown).

**Figure 1:**
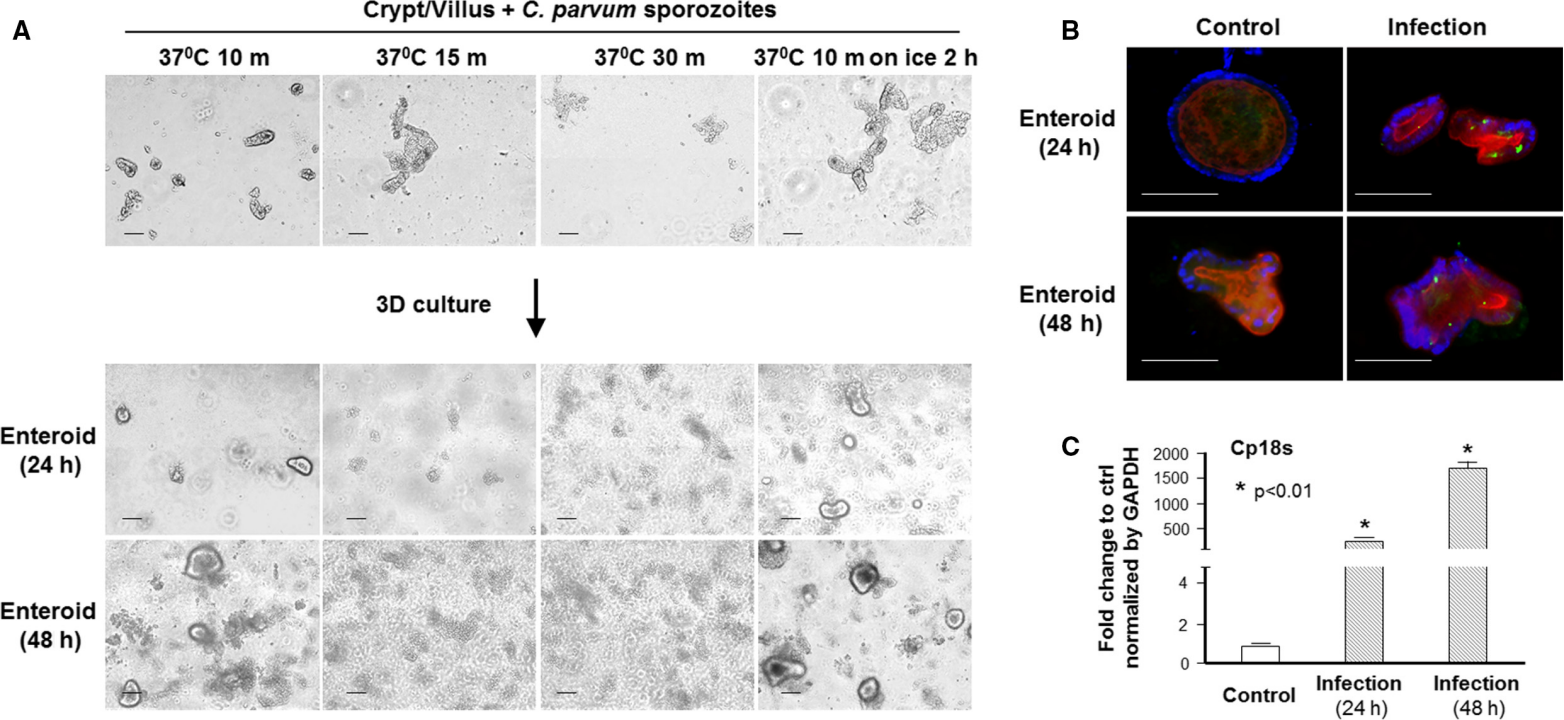
Infection of enteroids by *C. parvum* during propagation in culture. (A) Phase images of crypt/villus units or enteroids from adult mice following exposure to *C. parvum* sporozoites. Freshly excysted sporozoites were incubated with isolated crypt/villus units at different temperature conditions for various time periods followed by enteroids development in culture. Exposure to parasite for 10 min at 37°C and then for additional 2 h on ice did not show obvious effects on the morphological characters of the enteroids in 3D cultures. (B) and (C) Infection of enteroids from adult mice by *C. parvum* as evident by immunofluorescent staining and by quantification of Cp18s. Crypt/villus units immediately after isolation were exposure to freshly excysted sporozoites for 10 min at 37°C and then for additional 2 h on ice, followed by 3D cultures for 24 h and 48 h. Infection of the enteroids by *C. parvum* was shown by indirect immunofluorescent staining using an antibody specific to *C. parvum* (B) or quantified by real‐time PCR analysis of Cp18s (C). Data represent means ± SE from three independent experiments. * indicates a *P* < 0.01 between infection and noninfected control. Bar = 500 *μ*m.

We initially optimized the conditions for enteroids for *C. parvum* infection, using enteroids from adult mice. We first used these enteroids cultured for 24 h after isolation and exposed to *C. parvum* oocysts or excysted infective sporozoites for 8–24 h. These enteroids were collected from the dishes for infection assay either by immunofluorescent staining with a specific antibody to *C. parvum* (Chen et al. 2007; Zhou et al. 2012) or by real‐time PCR analysis of Cp18s (Zhou et al. 2012). No direct infection was observed either by immunofluorescent staining or by real‐time PCR analysis (data not shown), probably because that cultured enteroids were sealed and intact (Zachos et al. 2016) and the parasite could not access to the lumen to infect the enterocytes. This is also consistent with previous observations that *C. parvum* sporozoites can only infect the apical membrane surface of a polarized epithelium (Chen et al. 1998). We then exposed the crypt/villus units immediately after isolation to freshly excysted *C. parvum* sporozoites under different conditions, followed by 3D culture. We found that incubation of the crypt/villus units with the parasites for longer than 15 min at 37°C would significantly lower their viability, as observed under phase microscopy (Fig. 1A). Incubation for 10 min at 37°C and then for additional 2 h on ice did not show obvious morphological damage in the enteroids at 3D cultures (Figure 1A). Therefore, we applied this setting for all the experiments hereafter in this study unless otherwise described. Infection of the enteroids by the parasite was confirmed by immunofluorescent staining and real‐time PCR analysis of Cp18s (Figure 1B and 1C). Of note, the infection burden per cell under this experimental setting appears lower than that in cell cultures from previous studies using various intestinal epithelial cell lines (Zhou et al. 2012; Hu et al. 2013).

### Enteroids from neonatal mice for ex vivo *C. parvum* infection and from in vivo infected neonatal mice

**Figure 2:**
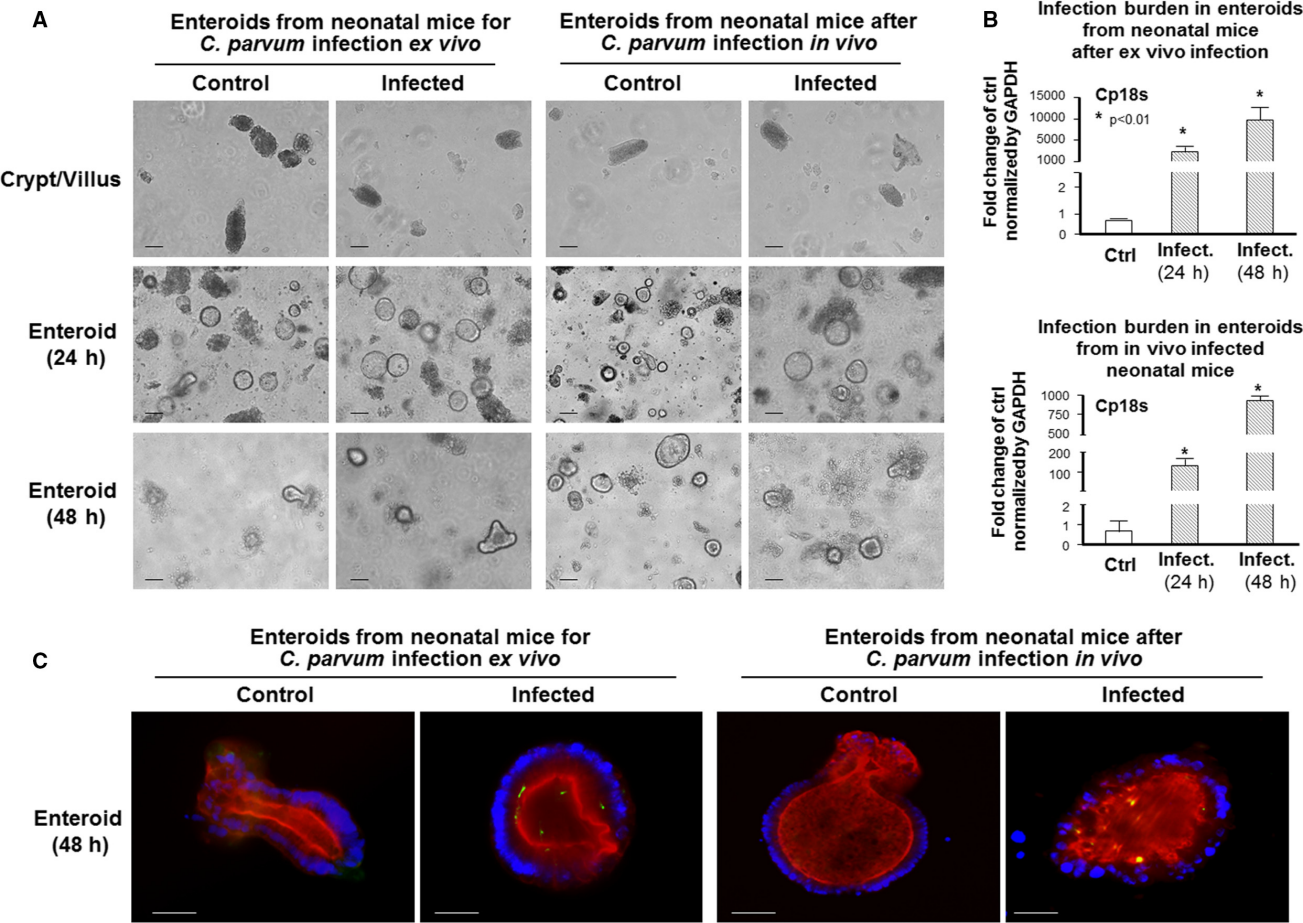
Infection of enteroids from neonatal mice by *C. parvum* ex vivo and by oral gavage administration in vivo. (A) Phase images of enteroids from neonatal mice infected by *C. parvum* ex vivo and by oral gavage administration in vivo. For ex vivo infection, crypt/villus units isolated from neonatal mice were exposed to *C. parvum* sporozoites and then cultured in 3D for 24 h and 48 h. For *C. parvum* infection in vivo, neonatal mice (*n* = 5) of 5–7 days old were given *C. parvum* oocysts by oral gavage. At 24 h after parasite challenge, crypt/villus units were isolated and cultured in 3D for additional 24 h and 48 h. (B) and (C) Infection of enteroids from neonatal mice by *C. parvum* ex vivo and in vivo as evident by immunofluorescent staining and by quantification of Cp18s. Infection of *C. parvum,* either in enteroids infected by *C. parvum* ex vivo or in enteroids from oral gavage infection in vivo, was quantified by real‐time PCR analysis of Cp18s (B) and shown by indirect immunofluorescent staining using an antibody specific to *C. parvum* (C). Data represent means ± SE from three independent experiments. * indicates a *P* < 0.01 between infection and noninfected control. Bar = 500 *μ*m.

Using crypt/villus units isolated from neonatal mice, we further characterized the infection of enteroids by *C. parvum* sporozoites. Taken the same approach as for the adult mice, crypt/villus units isolated from neonatal mice at 5–7 days after birth were exposed to infective *C. parvum* sporozoites and then cultured in 3D gel for enteroid propagation (Fig. 2A). Compared with that in enteroids from adult mice, a slightly higher infection burden was identified in the enteroids from neonatal mice as revealed by either immunofluorescent staining or real‐time PCR analysis of Cp18s (Fig. 2B and C).

We then isolated ileum crypto/villus units from *C. parvum* infected neonatal mice, using a well‐documented model of intestinal cryptosporidiosis in neonatal mice through oral administration (Novak and Sterling 1991; Lacroix et al. 2001). Different from the ex vivo infection approach described above, we cultured the isolated crypt/villus units immediately after separation from the intestine. Infection was evident by both immunofluorescent staining and real‐time PCR analysis of Cp18s (Figure 2B and 2C).

### Decreased bud formation in enteroids after ex vivo *C. parvum* infection

**Figure 3:**
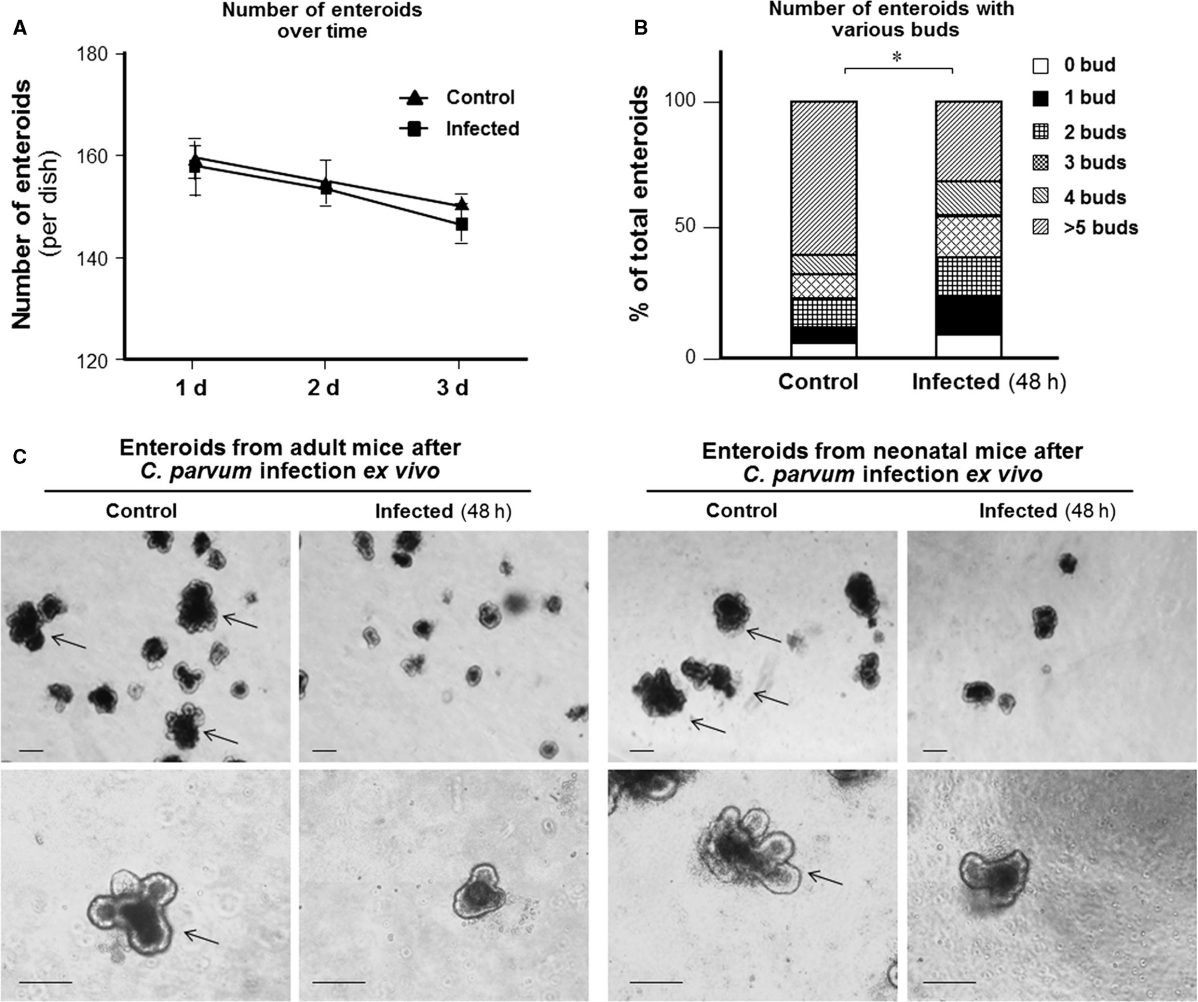
Decreased bud formation in enteroids after ex vivo *C. parvum* infection. (A) The total number of enteroids in the infected cultures remained no significant change over up to 48 h after infection, comparable with the enteroids number in the noninfected cultures. (B) Quantification of buds in enteroids from adult mice after ex vivo *C. parvum* infection for 48 h. The percentage of enteroids with various numbers of buds in the infected (*N* = 269) and noninfected control enteroids (*N* = 283) is shown. (C) Representative phase images showing a decreased bud formation in enteroids from either adult or neonatal mice after ex vivo *C. parvum* infection for 48 h. Whereas many buds were observed in the noninfected 3D cultured enteroids (indicated by arrows), only a few buds were detected in the enteroids after ex vivo *C. parvum* infection for 48 h. Data represent means ± SE from three independent experiments. * indicates a *P* < 0.01 between infection and noninfected control. Bar = 500 *μ*m.

We then asked whether infection may inhibit enteroids propagation or development. We first measured the number of enteroids at various time periods after exposure to *C. parvum*. No significant difference in the number of enteroids was observed between the infected cultures and noninfected control cultures (Fig. 3A). Enteroids growing in 3D culture recapitulate the cellular hierarchy of intestinal epithelium with these proliferative cells localized in crypt‐like “budding” compartments and differentiated nondividing cells into villus‐like regions at the enclosed central (Sato and Clevers 2013). We observed a decreased bud formation in enteroids from either adult or neonatal mice after ex vivo *C. parvum* infection for 48 h. Whereas many buds were observed in the noninfected 3D cultured enteroids, only a few buds were detected in the enteroids after ex vivo *C. parvum* infection for 48 h (Fig. 3B, shown data of enteroids from adult mice only). Accordingly, a significant lower percentage of enteroids with buds after ex vivo *C. parvum* infection was quantified in enteroids from either adult or neonatal mice after ex vivo *C. parvum* infection for 48 h, comparing with noninfected control (Fig. 3C).

### Decrease in the expression levels of ISC markers in enteroids following infection

**Figure 4:**
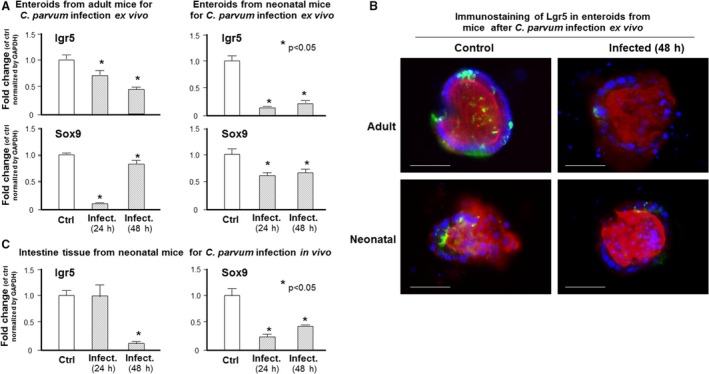
Expression levels of intestinal stem cell markers in enteroids following infection by *C. parvum*. (A) The expression levels of two intestinal stem cell markers, Lgr5 and Sox9, in enteroids following *C. parvum* infection ex vivo, as quantified by real‐time PCR. A significant decrease in the RNA levels of Lgr5 and Sox9 was detected in the enteroids from either adult or neonatal mice following *C. parvum* infection ex vivo, compared with the noninfected controls. (B) Decreased expression of Lgr5 at the protein level in enteroids following *C. parvum* infection ex vivo*,* as demonstrated by immunofluorescent staining with an antibody to Lgr5. (C) A decrease in the RNA levels of Lgr5 and Sox9 in the intestinal epithelium in neonatal mice following *C. parvum* infection in vivo through oral gavage. The ileum intestinal epithelium was collected from neonatal mice (*n* = 5) at 24 and 48 h after *C. parvum* oral gavage administration. RNA levels were compared with that in the ileum epithelium from noninfected animals. Data represent means ± SE from three independent experiments. * indicates a *P* < 0.05 between infection and noninfected control. Bar = 500 *μ*m.

Given our observation that a decreased bud formation was detected in enteroids following infection, we asked whether *C. parvum* infection of enterocytes may affect the function of stem cells in the crypts. We measured by real‐time PCR the expression levels of two intestinal stem cell markers, Lgr5 and Sox9, for the active and quiescent stem sub‐populations, respectively (Yan et al. 2012; Zhang and Huang 2013). We detected a significant decrease in lgr5 and Sox9 RNA levels in enteroids following infection (Fig. 4A). By immunohistochemistry, we observed that the number of Lgr5‐positive cells declined in infected enteroids, compared with uninfected control enteroids (Fig. 4B). Moreover, decrease in the RNA levels of Lgr5 and Sox9 was further confirmed in ileum intestinal epithelium isolated from in vivo infected neonatal mice after oral gavage of *C. parvum* oocysts (Fig. 4C).

### Alterations in gene expression levels in enteroids following *C. parvum* infection

**Figure 5:**
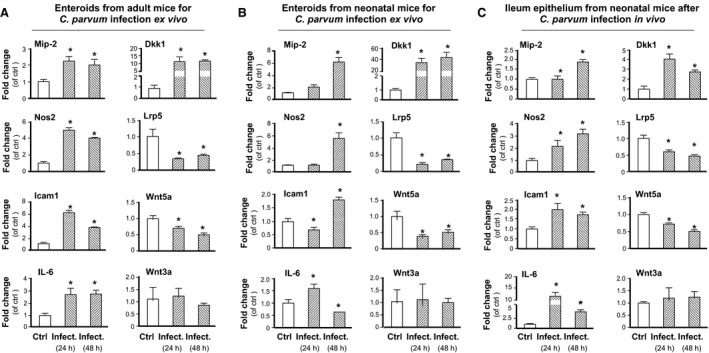
Alterations in the expression levels of selected genes in enteroids following infection by *C. parvum*. (A–C) The expression levels of selected genes were quantified by real‐time PCR. Genes measured were selected from previous studies demonstrating their alterations in host cells following *C. parvum* infection either in vitro or in vivo. Similar alterations in expression levels of selected genes were found in ex vivo infected enteroids from adult mice (A) and neonatal mice (B) and in ileum epithelium from neonatal mice following *C. parvum* infection in vivo through oral gavage (C). Experiments were in triplicate and data were represented in the RNA ratio to noninfected control as normalized to GAPDH. Data represent means ± SE from three independent experiments. * indicates a *P* < 0.01 between infection and noninfected control.

We further measured the expression levels of selected genes in enteroids following infection. Several inflammatory genes, including *Mip‐2, Nos2, Dkk1, Icam‐1,* and *IL‐6,* were measured by real‐time PCR, based on results from previous studies using various in vitro or in vivo models (Deng et al. 2004; Yang et al. 2009). Because the induction of Wnt/*β*‐catenin activation is a key factor for intestinal stem cell function (Clevers et al. 2014), expression levels of Dkk1 (a Wnt antagonist) (Chae et al. 2016), Wat3a and Wnt5a (two Wnt agonists) (Ishitani et al. 2003), and Lrp5 (a Wnt co‐receptor member) (Tamai et al. 2000), were also measured. Consistent with results from previous studies (Deng et al. 2004; Yang et al. 2009), we detected upregulation of *Mip‐2, Nos2, Dkk1, Icam‐1,* and *IL‐6* genes in the infected enteroids at 24 and 48 h after infection, compared with the noninfected control (Fig. 5A). Downregulation of *Lrp5* and *Wnt5a* genes was also observed in the infected enteroids (Fig. 5A), consistent with data of previous studies (Deng et al. 2004). No change in the expression level of *Wnt3a* was detected in infected enteroids (Fig. 5A). Alterations in the expression levels of these genes were observed in the enteroids from neonatal mice following *C. parvum* infection ex vivo (Fig. 5B) and in ileum epithelium from in vivo infected neonatal mice (Fig. 5C).

### Increased apoptosis and senescence of epithelial cells in enteroids following ex vivo *C. parvum* infection

**Figure 6:**
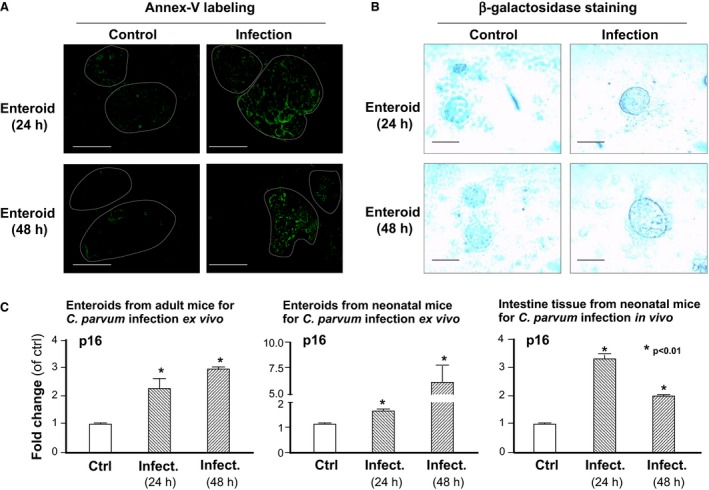
Apoptotic cell death and cellular senescence in enteroids induced by *C. parvum* infection. (A) Apoptotic cell death in enteroids from adult mice after ex vivo *C. parvum* infection for 24 h and 48 h, as measured by staining of Annexin‐V‐FITC. Representative fluorescent images from five independent experiments, along with phase images of the same fields, are shown. (B) Cellular senescence in enteroids after ex vivo *C. parvum* infection for 24 h and 48 h, as labelled by positive staining of *β*‐galactosidase. Representative images from five independent experiments, along with phase images of the same fields, are shown. (C) An increase in the expression level of p16, a cellular senescence marker, was also detected in enteroids after ex vivo *C. parvum* infection for 24 h and 48 h, as measured by real‐time PCR. Data represent means ± SE from three independent experiments. * indicates a *P* < 0.01 between infection and noninfected control. Bar = 500 *μ*m.

Previous studies have demonstrated apoptotic cell death of epithelial cells following *C. parvum* infection (Chen et al. 1998; Sasahara et al. 2003). At least at the early phase of the infection process, apoptotic cell death appears to be limited to the bystander noninfected cells (Chen et al. 1998). We then measured the apoptotic cell death in the enteroids from the adult mice during *C. parvum* infection using the Annexin‐V staining. A significant increase in the Annexin‐V positive cells was observed in the infected enteroids, compared with the noninfected control (Fig. 6A). Intriguingly, we detected a strong positive staining of *β*‐galactosidase, reflecting cellular senescence, in enteroids after ex vivo *C. parvum* infection for 24 and 48 h (Fig. 6B). Accordingly, we also detected an increase in the senescent cell marker, p16, in the enteroids after ex vivo *C. parvum* infection (Fig. 6C).

## Discussion

We found that to enable *C. parvum* to infect enteroids, the freshly excysted sporozoites need to be added to the culture shortly after the villus/crypts are isolated from the animals. This is expected because, after ex vivo culture and propagation, each enteroid develops as a functional unit with an intact but sealed lumen. At this point, *C. parvum* sporozoites would not be able to access the luminal surface to infect host cells. This is consistent with previous studies for other microbes and some of these studies used microinjection to deliver the pathogens into the lumen for apical infection (Foulke‐Abel et al. 2014). Analysis of gene expression profile in enteroids following ex vivo infection confirms these findings of previous studies in cultured enterocytes in vitro. Induced genes are often these inflammatory or defense genes, such as *Nos2*,* Mip2*, and *Icam1* (Yang et al. 2009; Deng et al. 2004). Such alterations in gene expression profile were further confirmed in the intestinal enteroids isolated from infected neonatal mice following oral gavage of *C. parvum* oocysts in vivo.

Immunocompetent mice are naturally resistant to *C. parvum* if given by oral gavage. We found that enteroids from adult mice are susceptible to ex vivo infection, suggesting that a systemic immune response and/or a mature intestinal microbiome may count for the resistant to infection in vivo in adult mice. Similar alterations in gene expression pattern induced by *C. parvum* were detected in enteroids from neonatal and adult mice. This suggests to us that the epithelial response to infection in neonatal and adult mice, in the absence of a systemic immune response and a mature intestinal microbiome, may be similar. However, a slight higher infection burden was detected in enteroids from neonatal mice than that in enteroids from adult mice, given the fact that the same amount of parasite sporozoites were initially added to the culture. Therefore, immature intestinal epithelium, such as in neonatal mice, may have deficiency in clearance of *C. parvum* infection. In this regard, enteroids as a model for intestinal cryptosporidiosis may provide a unique tool for further studies aimed to explore epithelial defense response against *C. parvum* infection.

One of the key findings of this study is the inhibitory effects of *C. parvum* infection on the ex vivo development of intestinal enteroids. Pathologically, *C. parvum* infection triggers a mild inflammatory infiltration and causes a shorter height of the intestinal villi in the ileum epithelium (Sasahara et al. 2003). Most importantly, we detected a significant decrease in the expression levels of intestinal stem cell markers, Lgr5 and Sox9 (Yan et al. 2012; Zhang and Huang 2013), in these enteroids following infection. Decrease in stem cell markers was also confirmed in the ileum epithelium from neonatal mice following oral gavage of the parasite. The intestinal epithelium exhibits a remarkable capacity of self‐renewal, renewed rapidly every 3–5 days in human, to maintain intestinal homeostasis (Barker et al. 2010); this property reflects the activity of multipotent intestinal stem cells which divide and differentiate to produce the different cell types that comprise the intestinal epithelium (Barker 2014). Lgr5^+^ cells and Sox9^+^ cells are two functionally different populations of intestinal stem cells: Lgr5 for the active ISC population and Sox9 for the quiescent ISC population (Yan et al. 2012; Zhang and Huang 2013). Both Lgr5^+^ and Sox9^+^ stem cell populations have mutipotency capabilities and are able to divide and later differentiate into all intestinal subtypes in the intestinal epithelium.

Increasing evidence supports that induction of Wnt/*β*‐catenin activation is a key factor for ISC function (Clevers et al. 2014). Since *C. parvum* infection is usually limited to enterocytes, we speculate that infected enterocytes may elicit unique signals that suppress function of the intestinal stem cells in the crypt. Interestingly, we detected a significant increase of Dkk1 and decrease of Wnt5a in the enteroids after infection, consistent with results from previous in vitro studies using multiple murine and human intestinal enterocyte cell lines for *C. parvum* infection (Deng et al. 2004; Yang et al. 2009). Functionally, Dkk1 is a Wnt antagonist (Chae et al. 2016) and Wnt5a is one of the major agonists for Wnt receptors (Ishitani et al. 2003). In addition, Lrp5, one of the Wnt co‐receptor members (Tamai et al. 2000), is downregulated in the infected enteroids. Moreover, Lgr5 is a Wnt target gene (van de Wetering et al. 2002) that marks proliferative stem cells in several Wnt‐dependent stem cell compartments, including the small intestine and colon (Barker et al. 2007). Decreased expression level of Lgr5 in infected enteroids may reflect the inhibition of Wnt/B‐catenin signaling in ISCs following *Cryptosporidium* infection. Functionally, Lgr5 has been demonstrated to interact with Wnt receptor Lrp5/6 and mediate ISC function in response to R‐spondin 1 (a potential Lgr5 legand) (de Lau et al. 2011; Ruffner et al. 2012). Therefore, it is feasible that *C. parvum* infection may inhibit ISC function through attenuation of the Wnt/B‐catenin signaling, a particular hypothesis currently undergoing investigation. Consequently, inhibition of ISC function will alter the differentiation of ISCs into various epithelial cell types and will eventually prohibit the turnover of intestinal epithelium, providing an obvious benefit to the parasite replication, as the life cycle of the parasite intracellular stage takes several days to complete (Chen et al. 2002).

Cell apoptotic death and senescence may also contribute to the negative impact of *Cryptosporidium* infection on intestinal epithelial growth. Apoptotic cell death and development of host cell senescence have been demonstrated in both in vitro and in vivo infection models in several parasites, including *T. cruzi*,* P. falciparum*, and *N. ceranae* (Deng et al. 2004; Carmen and Sinai 2007). Accelerated senescence was reported in human erythrocytes cultured with *P. falciparum* (Omodeo‐Salè et al. 2003). Membrane damage leading to accelerated senescence of both infected and uninfected erythrocytes may contribute to malaria anemia (Omodeo‐Salè et al. 2003). Apoptotic cell death associated with *C. parvum* infection has previously been reported in enormous studies (Sasahara et al. 2003), including these by ourselves (Chen et al. 1998). In this study, we detected the development of senescence in enteroids following *C. parvum* infection. Which epithelial cell types undergo senescence, what mechanisms are involved and what is its pathophysiological significance, merit further investigation.

In summary, we report a negative effect of *Cryptosporidium* infection on intestinal epithelial growth using an ex vivo model of intestinal cryptosporidiosis employing enteroids from neonatal and adult mice. Our findings not only support a novel ex vivo model of *C. parvum* infection in murine enteroids, but also reveal a potential function suppression of intestinal stem cells and development of host cell senescence after infection, providing additional insights into the impact of *Cryptosporidium* infection on intestinal epithelial growth.

## Conflict of Interest

The authors disclose no conflict of interest.
